# Primary Hyperparathyroidism due to Parathyroid Adenoma Originated from Supernumerary Gland

**DOI:** 10.1155/2018/6482546

**Published:** 2018-09-09

**Authors:** Fernando Mendoza-Moreno, Ángel Rodriguez-Pascual, María Rocío Díez-Gago, Marina Pérez-González, Laura Jiménez‐Alvárez, Isabel Furtado-Lobo, Manuel Díez‐Alonso, Fernando Noguerales-Fraguas

**Affiliations:** ^1^Department of General and Digestive Surgery, Príncipe de Asturias Teaching Hospital, Alcalá de Henares, Madrid, Spain; ^2^Department of Emergency Medicine, Príncipe de Asturias Teaching Hospital, Alcalá de Henares, Madrid, Spain

## Abstract

**Introduction:**

The variability of the location of the parathyroid glands is directly related to the events that occur during embryonic development. The impact that an individual submits more than four parathyroid glands is close to 13%. However the presentation of a parathyroid adenoma in a supernumerary gland is an uncommon event.

**Case report:**

A 30-year-old man diagnosed with primary hyperparathyroidism with matching findings on ultrasonography and scintigraphy for parathyroid adenoma localization lower left regarding the thyroid gland. A cervicotomy explorer showed four orthotopic parathyroid glands. The biopsy of the inferior left gland was normal. No signs of adenoma were seen in the biopsy. Following mobilization of the ipsilateral thyroid lobe, fifth parathyroid gland was found increased significantly in size than proceeded to remove, confirming the diagnosis of adenoma. After the excision, the levels of serum calcium and parathyroid hormone were normalized.

**Conclusions:**

The presentation of a parathyroid adenoma in a supernumerary gland is a challenge for the surgeon. The high sensitivity having different imaging techniques has been a key to locate preoperatively the pathological parathyroid gland. Analytical or clinical persistence of primary hyperparathyroidism after parathyroid surgery can occur if the location of the adenoma is a supernumerary or ectopic gland location.

## 1. Introduction

Primary hyperparathyroidism (pHPT) is the most frequent cause of increased serum calcium levels. The main cells of the parathyroid glands are responsible for secreting the parathormone (pHPT) which is involved in the reabsorption of calcium at the level of the distal tubule of the nephron, stimulating osteoclasts to promote bone resorption and catalyze the conversion of vitamin D3 in its active form [1].

The incidence of pHPT is 4 inhabitants per 1,000. It is twice as frequent as in postmenopausal women and in the fifth decade of life [2]. It is the most frequent cause of hypercalcemia. Among the etiology of pHPT, the parathyroid adenoma is the most frequent cause (80%) followed by other pathologies such as endocrine disorders of multiple neoplasia (MEN 1 and MEN 2A), hyperplasia of the parathyroid glands, or even carcinoma (1%) [3].

We present the case of a 30-year-old male with a history of primary hyperparathyroidism originated in a supernumerary parathyroid gland of ectopic location.

## 2. Case Report

A total of 376 patients diagnosed with pHPT were operated on in our service from January 2000 to December 2013.

We present the case of a 30-year-old male patient who referred to our clinic with the diagnosis of primary hyperparathyroidism. The patient had a history of stage IV-B non-Hodgkin lymphoma diagnosed in 2005 and treated with radiochemotherapy, currently in remission. Our patient presented with polydipsia and polyuria without associated bone pain. The laboratory tests showed a serum calcium of 12.7 mg/dl (laboratory range 8.7–10.4 mg/dl), phosphorus 2.4 mg/dl (2.4–5.1 mg/dl), hormone intact parathyroid (PTH) 216 pg/ml (11–80 pg/ml), 25-hydroxyvitamin D 23 ng/ml (30–100 ng/ml), alkaline phosphatase 102 U/l (45–129 U/l), 980 mg calciuria in 24 hours, and preserved renal function. Cervical ultrasound performed preoperatively showed a hyperechogenic nodule of 9 mm adjacent to the left lower thyroid pole. A scintigraphy with Tc99m sestamibi (MIBI) was performed in which a pathological hypercaptation was observed at the level of the left inferior thyroid.

**Figure 1:**
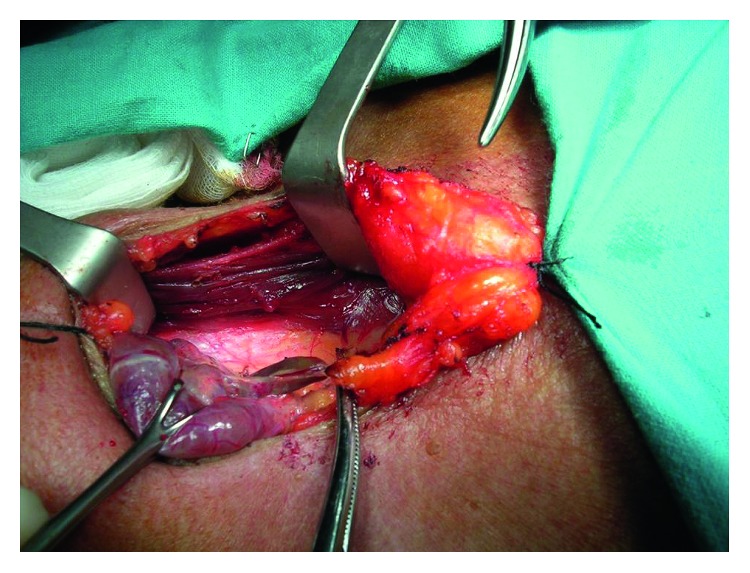
Parathyroid adenoma on the ectopic gland and its relationship with the left common carotid artery.

In view of these findings, the patient underwent an exploratory cervicotomy using a classic Kocher incision. During surgery, a normal-appearing thyroid parenchyma without nodules was observed. A slightly enlarged left lower parathyroid gland that was excised under the suspicion that adenoma was evidenced. The left superior gland was of normal appearance and macroscopic characteristics although a biopsy was performed. The intraoperative PTH levels did not decrease (PTH at the beginning of the surgery of 333 pg/ml, PTH after 20 minutes of its extraction of 366 pg/ml). The intraoperative result of both biopsies was of parathyroid tissue without histological alterations. It was decided to explore the right side finding, an upper and lower gland of normal macroscopic appearance and orthotopic localization. The left inferior thyroid lobe was mobilized, finding an extracapsular supernumerary gland adjacent to the common carotid artery with a maximum diameter of 15 mm that was excised (Figure 1). The decrease in PTH to 22.1 pg/ml and biopsy confirmed the diagnosis of a paratiorial adenoma.

The patient presented an immediate postoperative period normal, with evidence of a decrease in PTH and normalization of calcium levels. During twenty months of follow-up, the patient has not shown data of recurrence or persistence of hyperparathyroidism with calcemia and PTH within laboratory ranks.

## 3. Discussion

The first identification of the parathyroid glands was made by Sir Richar Owen in 1850 in the Indian rhinoceros [4]. Later, the medical student, Ivar Sandström, identified it on a corpse in 1877 [5]. However, it was not until the beginning of the 20th century, in 1925, when Félix Mandl performed the first parathyroidectomy, almost 30 years before the laboratory isolation of parathyroid hormone [6].

Approximately 80–97% of individuals have 4 parathyroid glands. About 2% of the subjects have 5 parathyroid glands.

Supernumerary parathyroid glands are called those glands coinciding in the same subject with four others. Its meaning is of clinical importance since it can be a cause of persistent hyperparathyroidism. Their number can vary between 5 and 11 as described by Akersröm in his series of 503 autopsies [7]. Its incidence is variable, presenting between 2.5 and 22%. Chiu et al. in their work consisting of the dissection of 160 corpses reported an incidence of 1.8% for individuals with 5 glands and 0.6% for those who had up to 6 parathyroids [8].

The supernumerary glands can be rudimentary, small, and close to a nonpathological gland (2%), divided, two very close glands divided by a thin septum of adipose tissue (6%), or separated from the rest without any of the above characteristics (13%) The rudimentary and divided glands may be a consequence of the stimulation of certain chronic situations such as uremia, reaching pathological significance.

Cervical exploration of the four glands has been considered as the gold standard in those patients who are going to undergo pHPT and whose location of the pathological gland does not show coincidences in the preoperative imaging tests. However, the increase in the sensitivity of some of them for their location has allowed us to perform a surgical approach aimed at performing a minimally invasive surgery consisting of selective cervicotomy with a small incision [9].

The previous history of exposure to radiotherapy in our patient may be related to the development of a posterior parathyroid adenoma. As with thyroid cancer, exposure to previous radiation is related to the development of hyperparathyroidism years later [10]. Rosen, in 1975, was the first to relate exposure to radiation with the development of tumors of the parathyroid gland [11]. It has been described that exposure to radiation in the head and neck or upper mediastinum is related to the development of a parathyroid adenoma up to 3 times more frequent than in the unexposed population [12]. This ratio is noticeably higher in patients exposed to high doses of radiation, such as that observed in workers who performed sealing tasks in the nuclear accident at Chernobyl in 1986 [10]. Woll et al. correlate both breast radiation with the development of a parathyroid adenoma and with the side on which it is located [13].

Among the imaging tests used for the preoperative localization of the adenoma, cervical ultrasound and MIBI are the most frequent. Cervical ultrasound, in addition to being an explorer-dependent test, represents the one with the lowest sensitivity (45–57%). MIBI, on the other hand, is usually useful for adenoma localization in most cases, reaching a sensitivity of up to 77–85% [14]. In our experience, we have 376 patients operated on for PPH from the year 2000 to 2013; these techniques had sensitivity similar to that reported in the published literature (cervical ultrasound sensitivity was approximately 37.5% and 71.5% for the MIBI). Some authors, like us, opt for a selective cervicotomy if both tests coincide in the location of the parathyroid adenoma while an exploratory cervicotomy is performed if they are discordant.

In our series, this case represents the first described case of parathyroid adenoma in a supernumerary gland with a cervical location. Only between 0.5–1% of the pHPT are by a fifth gland, and it is usually located in the mediastinum [3].

Currently, the MIBI is the test that is usually performed preoperatively. The performance of SPECT preoperatively in patients who are going to be operated on for the first time of PPH is controversial despite that the SPECT has a sensitivity close to 88%, and even combined with magnetic resonance imaging (MRI) or axial tomography (CT) (SPECT-NMR or SPECT-TC, respectively), it can reach values close to 92–94% [15–17]. In our series, it is not performed preoperatively, reserving the case for patients who, after surgery, experience persistence or recurrence of pHPT.

The use of intraoperative determination of PTH has been shown to be of great help in confirming the removal of pathological parathyroid tissue. For them, in all our patients, we have used the determination of PTH in three moments during the surgery (at the beginning, when visualizing the gland suggestive of parathyroid adenoma and after about 15 minutes of extirpating it) by applying the Miami criteria to refer to healing [16].

Finally, some authors like Norman and colleagues have described radioguided surgery for minimally invasive accesses. In our experience, it has been shown to be useful in those cases in which MIBI and cervical ultrasound were not coincided, and a selective approach was insisted by cervicotomy [17, 18].

## 4. Conclusions

The most frequent causes of persistent pHPT are the unidentified adenoma in the first surgical intervention, the parathyroid adenoma with ectopic location, an incomplete parathyroidectomy in cases of polyglandular disease (double adenoma or hyperplasia). Less common causes are local recurrence of an adenoma or parathyroid carcinoma, hyperplasia of a remnant after subtotal parathyroidectomy, or a fifth hyperplastic gland after an apparently complete subtotal parathyroidectomy.

The only curative treatment of pHPT is surgery consisting in the removal of the pathological gland. The clinical or analytical persistence of primary hyperparathyroidism after parathyroid surgery can occur if the location of the adenoma is a supernumerary or ectopic gland that during surgery had gone unnoticed. The presentation of a parathyroid adenoma in a supernumerary gland is rare and a challenge for the surgeon expert in thyroid and parathyroid surgery.

The high sensitivity presented by different imaging techniques such as MIBI or SPECT have been a fundamental tool to preoperatively locate the pathological parathyroid gland. In addition, techniques such as the intraoperative determination of PTH allow the biochemical confirmation of the complete removal of all hyperfunctioning parathyroid tissue, with less invasive surgeries, more effective, and with lower morbidity.
